# *Thuja occidentalis* L. (Cupressaceae): Ethnobotany, Phytochemistry and Biological Activity

**DOI:** 10.3390/molecules25225416

**Published:** 2020-11-19

**Authors:** Sonia Caruntu, Alina Ciceu, Neli Kinga Olah, Ioan Don, Anca Hermenean, Coralia Cotoraci

**Affiliations:** 1 Faculty of Pharmacy, Vasile Goldis Western University of Arad, Rebreanu 86, 310414 Arad, Romania; sonia.caruntu@gmail.com (S.C.); neliolah@yahoo.com (N.K.O.); 2 “Aurel Ardelean” Institute of Life Sciences, Vasile Godis Western University of Arad, Rebreanu 86, 310414 Arad, Romania; alina_ciceu@yahoo.com; 3 “Pavel Covaci” University Botanical Garden from Macea, Vasile Goldis Western University of Arad, Rebreanu 86, 310414 Arad, Romania; don_ioan@yahoo.com; 4 Faculty of Economics, Computer Science and Engineering, Vasile Godis Western University of Arad, Rebreanu 86, 310414 Arad, Romania; 5 Faculty of Medicine, Vasile Goldis Western University of Arad, Rebreanu 86, 310414 Arad, Romania; ccotoraci@yahoo.com

**Keywords:** *Thuja occidentalis* L., thujone, ethnobotany, pharmacology, biological activities

## Abstract

*Thuja occidentalis* L. (Cupressaceae) has its origins in Eastern North America and is cultivated in Europe and Brazil as an ornamental tree, being known as the “tree of life” or “white cedar”. In traditional medicine, it is commonly used to treat liver diseases, bullous bronchitis, psoriasis, enuresis, amenorrhea, cystitis, uterine carcinomas, diarrhea, and rheumatism. The chemical constituents of *T. occidentalis* have been of research interest for decades, due to their contents of essential oil, coumarins, flavonoids, tannins, and proanthocyanidines. Pharmacology includes antioxidant, anti-inflammatory, antibacterial, antifungal, anticancer, antiviral, protective activity of the gastrointestinal tract, radioprotection, antipyretic, and lipid metabolism regulatory activity. Therefore, the present review represents the synthesis of all the relevant information for *T. occidentalis*, its ethnobotany, phytochemistry, and a thorough analysis of their pharmacological activities, in order to promote all the biological activities shown so far, rather than the antitumor activity that has promoted it as a medicinal species.

## 1. Introduction

The approach of various complementary therapies is becoming an increasingly used method of treatment. In this context, the use of plants for the treatment of various diseases plays a major role [1,2,3]. Folk medicine is widely used in much of the world, even though it is not officially recognized in many countries [4]. Over 80% of the population in Africa and Asia use plants and plant extracts for the treatment of various diseases [5].

The use of phytomedicine began in China during the Xia dynasty and in India during the Vedic times. Herbal remedies are growing in popularity around the world for several reasons: They have long-lasting curative effects and are characterized by effectiveness, safety, and low side effects [6,7]. Currently, ethnopharmacological studies are encouraged worldwide as a method for highlighting species containing molecules or beneficial products that can be used in the pharmaceutical, dietary, and cosmetic fields [8,9]. The use of medicinal plants has increased significantly in Western countries due to the adverse effects of chemical drugs and significant financial benefits. Although it is difficult to estimate the exact amount earned from the trade in plants and plant extracts, it is certain that the pharmaceutical industry based on folk medicine grows annually by over 4% [10,11].

Medicinal plants are a source of bioactive natural compounds with high therapeutic potential. Today, biologically active compounds in plants and their derivatives are found in a high percentage in drugs on the pharmaceutical market. It is estimated that about 25% of current drugs are composed of natural compounds [12]. Moreover, more and more food supplements and cosmetics contain various plant extracts and biologically active compounds as main active ingredients [13].

The importance of medicinal plants is due to the great diversity of bioactive molecules with beneficial effect, as well as phenolic compounds, carotenoids, tocopherols, and vitamins. Due to their antioxidant properties, bioactive compounds are increasingly being studied for their beneficial effects against many chronic conditions, such as obesity, diabetes, and cancer [14,15,16,17]. Some bioactive compounds react slowly and need a long time to exert their antioxidant action. Moreover, the synergism between biomolecules in a mixture makes the target effect not only dependent on concentration, but also on the interaction between antioxidants [18,19]. In recent years, there has been an increase in the number of preclinical and clinical studies aimed at testing the use of plant extracts and biologically active compounds with beneficial effects on human health [20].

Thuja trees belongs to the order Pineales, in the *Cupressaceae* family [21]. The Thuja genus contains five species: *Thuja koraiensis*, Nakai-Korean thuja; *Thuja occidentalis* L., eastern arborvitae, northern white cedar; *Thuja plicata* Donn ex D. Don, Western red cedar; *Thuja standishii* (Gordon), Carrière-Japanese thuja; and *Thuja sutchuenensis*, Franch-Sichuan thuja. They are evergreens trees growing from 3 to 60 m tall with flattened branchlets, distributed in North America and East Asia.

The leaves of *T. koraiensis* have a high content of vitamin C, being used by the American natives and the first European explorers as a treatment for scurvy. The leaves of this tree have also been used as a treatment for rheumatism [22]. *T. koraiensis* oil is a known remedy with topical application in the treatment of the human papilloma virus (HPV), as well as for genital or common warts treatment [23], as an antioxidant [24], and for antibacterial properties [22].

Due to its antimicrobial and insecticidal properties, *T. plicata* essential oil has traditionally been used for wood preservation and insect repellent [25,26,27]. *T. plicata* leaf oil has traditionally been used by Aborigines in the Pacific Northwest, to treat a number of upper respiratory tract diseases [25]. Moreover, its essential oil has antibacterial and antifungal activity [26]. Its antimicrobial effect is thought to be due to the high concentration of α- and β-thujone, the main biologically active compounds in various essential oils that possess similar antimicrobial properties [28,29].

*T. standishii* is also used for its medicinal properties for the treatment of Epstein-Barr virus [30,31] and for antitumor activity [32].

The summary of the bioactive potential of the *Thuja* species, according to its applications, is presented in Table 1.

The present work provides an overview about the state-of-the-art in ethnobotany, phytochemistry, and a detailed analysis of the pharmacological properties of *T. occidentalis*, such us: antioxidant, anti-inflammatory, antibacterial, antifungal, anticancer, antiviral, protective activity of the gastrointestinal tract, radioprotection, antipyretic, and lipid metabolism regulatory activity, in order to promote all the biological activities shown so far, rather than the antitumor activity that has promoted it as a medicinal species.

## 2. Ethnobotany

**Figure 1:**
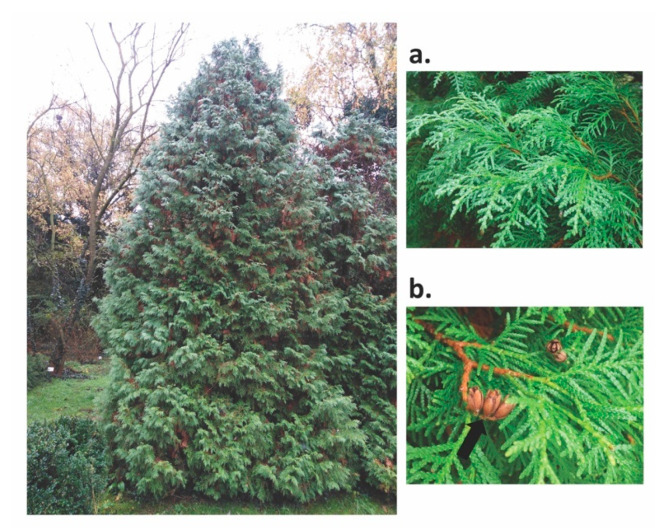
Specimen of *T. occidentalis* from the “Pavel Covaci” University Botanical Garden of Macea, Romania; detail: twig of leaves showing the flattened branches (**a**) and small pins containing the seeds (**b**).

*Thuja occidentalis* originated in the Eastern North America and is cultivated in Europe and Brazil as an ornamental tree [57,58,59], known as the “tree of life” or “white cedar” (Figure 1) [21].

In traditional medicine, *T. occidentalis* has been used in the treatment of liver diseases, bullous bronchitis, psoriasis, enuresis, amenorrhea, cystitis, uterine carcinomas, diarrhea, and rheumatism [58,60,61,62]. Essential leaf oil was used in the treatment of fungal infections, cancer, and intestinal worms [41]. As a tincture, *T. occidentalis* has been used in the treatment of warts, papillomas, and condylomas caused by human papilloma virus (HPV) [57,63].

Since historical times, the essential oil of *T. occidentalis* has been used in folk medicine. *T. occidentalis* containing thujone was used for hepatoprotection, bronchial catarrh, rheumatism, psoriasis, and even uterine carcinomas.

The mother tincture of *T. occidentalis* is used in homeopathy against a number of diseases. Homeopathy uses thuja as one of the main remedies for psychotic constitutions, in case of snake bite, small-pox, and vaccination-induced toxicity, respectively the proliferation or pathological vegetation [64].

Thuja preparations with thujone were regularly used by the American Indian traditional healers. The decoct and the tea prepared from the inner bark of the unwoody twigs could relieve constipation and headache [65]. It has also been used for the treatment of polyps, birthmarks, and wounds and as a painkilling and anthelmintic remedy [66,67].

## 3. Phytochemistry

The chemical composition of the *Thuja occidentalis* is summarized in Table 2.

The fresh plant contains approximately 0.6% essential oil (EO), 2.07% reducing sugars, 4.9% polysaccharides, 2.11% minerals, 1.67% free acids, and 1.31% tannic agents [68]. The main monoterpenes identified in the EO obtained from fresh leaves are 65% thujone, 8% isothujone, 8% fenchone, 5% sabines, and 2% α-pinene [68]. Other identified monoterpenes are: carvotanacetone, origanol, origanes, myrcen, and camphen [68,69,70,71].

Moreover, high molecular weight glycoproteins or polysaccharides have been shown to be particularly relevant for plant activity [72].

The dry plant has 1.4–4% EO, whose constituents are borneol, camphene, fenchone, limonene, myricene, α-terpine, terpinolene, thujone, thujylalcohol. Thujone consists of 85% α-thujone and 15% β-thujone is the main compound (0.76–2.4%) found in the EO obtained from the dry plant. Other compounds present in the dry plant are coumarins, represented by *p*-coumaric acid and umbelliferone; flavonoids, e.g., kaempferol, kaempferol-3-*O*-α-rhamnoside, mearnsitrin, myricetin, myricitrin, quercetin, and quercitrin; and tannins, (+/−)-catechins, (−)-gallocatechin, and proanthocyanidines, like pocyanidin B-3 and prodelphinidin.

Other constituents are tannic acid, which is about 1.3%, as well as polysaccharides and proteins representing approximately 4% [58,68,73,74,75,76,77,78]. Nazir et al. (2016) have found that the *T. occidentalis* leaf methanolic extract contains 135.32 mg/g total polyphenols expressed in gallic acid equivalent and 3.46 mg/g of total flavonoids expressed in quercitin equivalent [36].

Thujone is the monoterpene found in the composition of many plants including *Thuja occidentalis*. However, the use of this compound is regulated by the European Parliament and Council and the European Medicines Agency [67]. The isomers α-thujone and β-thujone are monoterpene ketones, with the following IUPAC name: (1*S*,4*R*,5*R*)-4-methyl-1-(propane-2-yl) bicyclo (3.1.0) hexan-3-one [67].

In a previous study, we found, in an ethanolic extract made from fresh leaves, 21.13 μg/mL thujone, 2.16 mg/mL total phenolic acids expressed in caffeic acid, 0.36 mg/mL total flavonoids expressed in rutoside, respectively [37].

However, to date, the genetic regulation of thujone synthesis pathway has been studied in two species: *Salvia officinalis* and *Thuja plicata*. Geranyldiphosphate (GPP) and neryl-diphosphate are the general precursors’ starting points and follow a four-step pathway. Sabinene is the first monoterpene in this path, being catalyzed by the enzyme sabinene-synthase (SS). It has been showed that sabinene is the precursor of thujone in *S. officinalis*, *A. absinthium*, and *T. vulgare*. Next, in three consecutive steps, thujone is formed from sabinene. The next intermediary in thujones’ synthesis is sabinone, from which begins the thujone synthesis. There are some corresponding enzymes of this transformation which have not yet been clarified and also the formation of the two isomers of thujone (α and β). There are some species who get both isomers, while other plants accumulate just one of them [67].

## 4. Biological Activities

*Thuja occidentalis* L. presents a varied range of pharmacological activities, such as antioxidant activity [36,38], anti-inflammatory [21], antibacterial, antifungal [26,33,79,80,81,82], antitumoral [42,43,44,63,83,84,85], antidiabetic [47], hypolipidemic, antiatherosclerotic [48], gastroprotective [45], antiviral, immunostimulent [73,74,75], radioprotector [49,86], and sedative [87]. Pharmacological and clinical investigations have started to use the effects of thujone biological activities.

### 4.1. Antioxidant Activity

The antioxidant capability is the best described property of phenolic compounds toward free radicals which are produced by cells metabolism [88].

In our previous studies, we showed a high antioxidant activity of a mother tincture of *Thuja occidentalis* by 2,2-diphenyl-1-picrylhydrazyl radical (DPPH) containing (3.9 mg GAE/g d.w.) polyphenols, as well as its in vitro antioxidant capacity on Caco-2 cells exposed to oxidative stress induced by H_2_O_2_, by MDA- and GSH-level assessments [37].

Nazir et al. (2016) showed that the antioxidant potential of the *Thuja occidentalis* methanolic extract was assumed to high DPPH radical scavenging activities, ABTS, NO and lipid peroxidation assays [36].

In their study, Mighri et al. (2010) examined the antioxidant and antimicrobial activity of four essential oil types, whose major constituents were β-thujone, α-thujone, α-thujone/β-thujone, and 1,8-cineole/camphor/α-thujone/β-thujone, and the composition was investigated by using capillary GC and GC/MS technique. The results of the study showed that all examined oils had a great antimicrobial potential, and, in addition, they assessed the antioxidant capacity by different in vitro tests [89]. Moreover, in a study, Mahomoodally et al. (2019) assessed the antioxidant potential, antiglycation, and the total phenolics content of essential oils extracted from 19 medicinal plants [90].

The antioxidant potential of *T. occidentalis* L. cones was assessed in raw chicken ground meat during refrigerated storage in a study made by Yogesh et al. (2014). DPPH free-radical scavenging activity method was used to estimate the antioxidant activity of thuja cones (*T. occidentalis*) and peach seeds (*Prunus persica*). They also estimated the total phenolics, flavonoids, and reducing power in these extracts. Thuja cones extract and peach seeds extract had a total phenolics of 7.80 ± 0.04 and 1.92 ± 0.04 mg TAE/g d.w., respectively [39].

Moreover, there has been a remarkable DPPH radical scavenging activity shown by both extracts (25.52 ± 1.92% and 24.99 ± 0.32%). However, thuja cones extract showed a better reducing power, as compared to peach seeds extract (3.32 ± 0.01 and 0.49 ± 0.01) [39].

### 4.2. Anti-Inflammatory Activity

Inflammation is a primary protective reaction that is given to return the damaged tissue due to injurious stimuli. This response involves extravasation of neutrophils through the capillary network and then activation of macrophages, which generate different proinflammatory cytokines, such as TNF-α, interleukins, and interferons, which participate in the regulation of inflammatory reactions. There are not many studies that have evaluated the anti-inflammatory activity of *T. occidentalis* components. The study made by Silva et al. (2017) highlighted the anti-inflammatory activity of the aqueous extract and the polysaccharide fraction obtained from *T. occidentalis* in experimental models of acute inflammation. The doses used were 3, 10, and 30 mg/kg, administered intraperitoneally. They acted through mechanisms that involve modulating mediators, such as histamine, serotonin, PGE2, and bradykinin, and diminishing vascular permeability and neutrophil migration to the affected site. The aqueous extract and the polysaccharide fraction of *T. occidentalis* reduced production of pro-inflammatory cytokines (TNF-α and IL-6), diminished both COX-2 and iNOS activity, and diminished oxidative stress. High doses of aqueous extract and fraction of polysaccharides obtained from *T. occidentalis* of 300 mg/kg did not result in gastric toxicity [21].

Dubey and Barta (2009a) highlighted the antioxidant activity of the ethanolic fraction (EFTO) of *T. occidentalis* in rats. EFTO inhibited lipid peroxidation induced by FeSO_4_ at doses of 100, 150, 200, 250, and 300 μg EFTO [38].

Polysaccharides obtained from thuja leaf extract have been shown to reduce mice-induced inflammation. They have the ability to prevent metastasis by diminishing inflammatory cytokines, such as IL-1β, IL-6, granulocyte-macrophage colony stimulating factor (GM-CSF), and TNF-α. Moreover, these polysaccharides stimulated the activity of natural killer (NK) cells, cell mediated antibody-dependent cytotoxicity (ADCC) and complement-mediated cytotoxicity (ACC) and stimulated the activity of antitumor factors, IL-2, and TIMP [43].

### 4.3. Antibacterial and Antifungal Activity

*T. occidentalis* has been shown to have antibacterial properties against a significant number of species, such as *Salmonella* sp., *Enterobacter cloacae*, *Staphylococcus aureus*, *Escherichia coli*, *Pseudomonas aeruginosa*, *Klebsiella pneumonia*, *Shigella flexenari*, *Candida albicans*, *Proteus vulgaris*, *Entercoccus faecalis*, and *Staphylococcus* [79,80]. They observed that the two components, α-thujone and β-thujone, showed protective effect against Gram-negative bacteria such as *Pseudomonas aeruginosa* and *Klebsiella pneumonia* and a mild protective effect against *Staphylococcus aureus*, *Escherichia coli*, and *Candida albicans* [80]. *Thuja occidentalis* also showed significant antibacterial activity against bacteria and fungi [79]. The antimicrobial profile of essential oil of *Thuja occidentalis* was also evidenced by Tsiri et al. (2009) [26].

The antifungal properties of *T. occidentalis* have been highlighted against *Saccharomyces cerevisiae*, *Aspergillus parasitious*, *Aspergillus niger*, *Aspergillus flavus*, *Trichophyton rubrum*, *Macrophomina*, and *Fusarium solani* [81,82]. In the recent study made by Bellili et al. (2018), essential oil extracted from leaves and cones of *Thuja occidentalis* showed antimicrobial activity against Gram-negative bacteria (*Escherichia coli*, *Salmonella typhimurium*, *Aeromonas hydrophila*, and *Pseudomonas aeruginosa*), Gram-positive bacteria (*Staphylococcus aureus*, *Listeria monocytogenes*, and *Bacillus cereus*), fungus (*Aspergillus flavus* and *Aspergillus niger*), and yeast (*Candida albicans*) [33].

### 4.4. Antiviral Activity

Polysaccharides isolated from *T. occidentalis* have been shown to have antiviral and immunostimulating effect, having the ability to inhibit HIV-1 and influenza A [34,91]. *Thuja polysaccharides* (TPS) inhibited HIV at a concentration of 625 μg/mL. They have been shown not to be toxic to MT-4 cells and have inhibited the expression of HIV-1 specific antigen in newly infected MT-2 cells [91]. Later, Gohla et al. (1992) highlighted the property of the high-molecular-weight polysaccharide fraction from *T. occidentalis* on HIV-1 [34]. An isolated fraction of *T. occidentalis* was shown to increase the number of cells producing antibodies in an in vitro study [86].

### 4.5. Anticancer Activity

In homeopathy, *T. occidentalis* is used in the treatment of cancer, but its mechanism of action is not known. Torres et al. (2016) studied the effect of α/β-thujone on glioblastoma, using in vitro and in vivo models. They have observed that α/β-thujone has the ability to diminish cell viability and has antiproliferative, proapoptotic, and antiangiogenic properties in vitro. In in vivo studies, α/β-thujone has been reported to induce regression of neoplasia and inhibited angiogenic markers of VEGF, Ang-4, and CD31 inhibitors in the tumor [42]. The antitumoral effect of the extract obtained from thuja leaves has been evaluated on numerous cancer cell lines [41,44,63,84].

The in vivo study performed by Siveen and Kuttan (2011b) demonstrated the antitumor effect of thujone in the malignant ascites lymphoma model (Dalton). Thujone, a monoterpene naturally found in *T. occidentalis*, has been shown to increase the number of leukocytes and bone marrow cells. This increased the proliferation of splenocytes and thymocytes, both in the presence and absence of specific mitogenes. Thujone stimulated cell mediated immune response and production of IL-2 and IFN-γ [92]. Thujone also showed the ability to inhibit metastasis in melanoma in vivo [83]. Thujone obtained from *T. occidentalis* ethanolic extract has been shown to have anticancer properties on the malignant melanoma cell line A375. In the same study, thujone was shown to have an antiproliferative effect and the ability to induce apoptosis [41].

Thuja’s antitumoral activity has been highlighted in breast cancer [85]. Polysaccharides obtained from *T. occidentalis* L. leaf extract have been shown to reduce mice-induced inflammation. They have the ability to prevent metastasis by diminishing inflammatory cytokines, such as IL-1β, IL-6, granulocyte-macrophage colony stimulating factor (GM-CSF), and TNF-α. Moreover, these polysaccharides stimulated the activity of natural killer (NK) cells, cell-mediated antibody-dependent cytotoxicity (ADCC), and complement-mediated cytotoxicity (ACC) and stimulated the activity of antitumor factors, IL-2, and TIMP [43].

### 4.6. Protective Activity of the Gastrointestinal Tract

According to Dubey and Batra (2008b), the ethanol fraction of *T. occidentalis* showed a hepato-protective effect in acute and chronic liver-induced HCV. The same researchers revealed that the ethanolic fraction of *T. occidentalis* provides an important effect against gastric lesions [46].

Saeed et al. (2014) evaluated the effect of low dose of *T. occidentalis* on rabbit, for three months. They carried out an experiment on rabbits, on treated groups with and without tetrachloride. They analyzed the heart, liver, stomach, and kidney tissues histopathologically, on both control groups and *T. occidentalis* treated groups. They also tested the CCl_4_ injected group by doing the liver function test. Their study showed that there were minor harmful effects in the liver and kidney tissues which were treated with carbon tetrachloride, but there was no major toxicity due to the antioxidant effect of active constituents from *T. occidentalis* [93].

The methanolic extract obtained from *T. occidentalis* orally administered at 200 mg and 400 mg/kg body weight has been shown to have gastroprotective effect in rats, comparable to omeprazole. This extract reduced gastric acid production by 45% and 69%, respectively, and favored a significant regeneration of the gastric epithelium at a dose of 400 mg/kg body weight. The antiulcer action of this extract is due to its antioxidant properties [45].

Our previous results show that orally administration of *T. occidentalis* mother tincture by gavage, for one week, to mice with experimentally induced ulcerative colitis, succeeded in inhibiting the inflammatory process induced by TNBS in the intestine, and normalized the structure and ultrastructure of the intestinal mucosa [37].

### 4.7. Lipid Metabolism Regulation

The ethanolic fraction of *T. occidentalis* has been shown to have hypoglycemic properties in rats with aloxan-induced diabetes, at a dose of 200 mg/kg, without significant impact on body weight. It has also improved lipid profile and has been shown to have a protective effect against oxidative stress by increasing glutathione level in blood [47].

In the study conducted by Dubey and Batra (2009a), the *T. occidentalis* ethanolic fraction administered at doses of 200 mg and 400 mg/kg body weight showed 77–92% decrease in serum cholesterol, with 53–84% LDL-cholesterol and 27–46% of triglycerides. Antiatherosclerotic activity was marked by the increase in HDL-cholesterol and the reduction in the atherogenic index. *Thuja occidentalis* showed a significant free-radical neutralization effect due to its ability to interfere with the absorption, degradation, and excretion of cholesterol [48].

### 4.8. Radioprotective Activity

*T. occidentalis* induced increased activity of TNF-α, IL-6, and IL-1 and exhibited a protective effect against radiation [94].

In the study made by Sunila and Kuttan (2005), *T. occidentalis* showed a protective effect against gamma-induced toxicity in Swiss albino mice. Thus, the *Thuja occidentalis* alcoholic extract reduced levels of alkaline phosphatase, pyruvate transferase, and lipid peroxidation [49].

### 4.9. Antipyretic Activity

The methanolic extract of *Thuja occidentalis* showed antipyretic activity in rabbits. It reduces fever and normalizes body temperature at doses of 100 mg and 200 mg/kg body, comparable to paracetamol [50].

The results of the in vitro and in vivo studies regarding the biological activities exerted by *T. occidentalis* are summarized in Table 3.

## Figures and Tables

**Table 1:** Comparison of the bioactive potential of *T. occidentalis* with other *Thuja* genus species.

Thuja Species	Benefits According to Biological Activities	
	Medicinal Use	Industrial Use
*T. koraiensis*	antimicrobial [22,23]; antioxidant [24]	-
*T. occidentalis*	antimicrobial [26,33,34,35]; antioxidant and anti-inflammatory [33,36,37,38,39,40]; antitumor [41,42,43,44]; hepatoprotective and gastroprotective [45,46]; antidiabetic [47]; antiatherosclerotic [48]; radioprotective [49]; antipyretic [50]	antifungal agent for biocontamination control in libraries and archives storage areas [51]; insecticidal activity [52]
*T. plicata*	antimicrobial [25,26,53]; anti-inflammatory, immunomodulatory, and tissue remodeling [54]	antimicrobial agent for decontamination of buildings [25]
*T. standishii*	antimicrobial [30,31,32]; antitumor [30,31,32]	-
*T. sutchuenensis*	antimicrobial [55,56]	-

**Table 2:** The chemical composition of the fresh and dried *T. occidentalis*.

**Chemical Composition of the Fresh Plant**	
Essential oil (*v*/*w*) Essential oil compounds (mainly monoterpenes): thujone (65%); isothujone (8%); fenchone (8%); sabines (5%); α-pinene (2%)	0.6%
Reducing sugar	2.07%
Minerals	2.11%
Free acids	1.67%
Tannic agents	1.31%
**The Constituents of the Dried Herbal Substance (*Thuja occidentalis Herbal*)**	
Essential oil	Borneol Camphene Fenchone Limonene Myricene α-Terpine Terpinolene Thujone (85% α-thujone and 15% β-thujone) is the main compound (0.76–2.4%)
Coumarins	*p*-Coumaric acid Umbelliferone
Flavonoids	Kaempferol Kaempferol-3-*O*-α-rhamnoside Mearnsitrin Myricetine Myricitrin Quercetin Quercitrin
Tannins	Catechine Gallocatechine
Proanthocyanidines	Procyanidin B-3 Prodelphinidin

**Table 3:** Biological activities of the *Thuja occidentalis* shown by in vitro and in vivo studies.

Biological Activity	In Vitro Studies	In Vivo Studies		Ref.
		Animal Model	Effects	
Antioxidant	↑ DPPH, NO, O2(-), ABTS scavenging activity ↑ Anti-LPO activity	-	-	[33,36,38,39]
	↑ DPPH and NO scavenging activity ↓ MDA, ↑ GSH	TNBS-induced colitis mouse model	↓ MDA, ↑ GSH	[37]
Anti-inflammatory		TNBS-induced colitis mouse model	↓ IL-6, TNF-α expression ↓ COX-2	[37,40]
Antibacterial	↓ Gram-negative/positive bacteria			[33]
Antifungal	inhibitory activity against the fungi causing keratitis			[35]
Antiviral	inhibition of HIV-1			[34]
Anticancer	↑ ROS generation ↑ Cyt c and caspase-3 activation ↑ DNA fragmentation ↑mitochondrial transmembrane potential collapse proapoptotic potential in the skin cancer cell line A375			[41]
	antiproliferative, proapoptotic and antiangiogenic properties ↓ tumor progression	Sprague-Dawley rats	↓ tumor size inhibition of angiogenic markers	[42]
		B16F-10 melanoma cells in mice	↓ IL-1β, IL-6, GM-CSF, TNF-α ↑ IL-2 and TIMP	[43]
	-	B16F-10 melanoma cells in C57BL/6 mice	inhibition of lung metastasis ↓ tumor-nodule formation ↓ lung collagen hydroxyproline ↓ lung uronic acid ↓ lung hexosamine ↓ serum sialic acid ↓ serum GGT	[44]
Hepatoprotective	-	Acute and chronic CCl_4_-induced rats liver damage	preserve normal histoarhitecture	[46]
Gastroprotective	-	acute gastric ulcer model in rats	antiulcer action regeneration of the gastric epithelium ↓ ulcer index ↓ gastric acid production	[45]
Antidiabetic	-	lloxan- induced diabetes in rats	hypoglycemic ↑ GSH improve lipid profile	[47]
Antiatherosclerotic	-	cholesterol fed rats	↓ cholesterol, LDL-cholesterol and triglycerides ↑ HDL-cholesterol ↓ atherogenic index free radical neutralization	[48]
Radioprotective	-	exposure of Swiss albino mice to γ-rays	↓ alkaline phosphatase ↓ glutamate pyruvate transaminase ↓ lipid peroxidation	[49]
Antipyretic	-	TAB vaccine-induced pyrexia models in albino rabbits	↓ fever normalized body temperature	[50]

Legend: 2,2-diphenyl-1-picrylhydrazyl radical (DPPH); NO—nitric oxide; ABTS-(2,2-azino-bis-3-ethyl benzthiazoline-6-sulphonic acid); TNBS-2,4,6-trinitrobenzene sulfonic acid; LPO—lipid peroxidation; MIC—minimum inhibitory concentration; MBC—minimum bactericidal concentration; GGT—gamma glutamyl transpeptidase; GPT—glutamate pyruvate transaminase; TAB (Typhoid); cyt C—cytocrome C, ↓ increase, ↓ decrease.
